# Complete thoracoscopic enucleation of the esophageal leiomyoma at the level of the azygos vein: A case report

**DOI:** 10.1016/j.ijscr.2021.106537

**Published:** 2021-10-25

**Authors:** Duc Nhan Le, Vinh Hieu Nguyen, Thi Nga Duong, Trong Vu Than

**Affiliations:** The Thoracic Surgery Department, Da Nang Hospital, Viet Nam

**Keywords:** Complete thoracoscopic enucleation, Esophageal leiomyoma, Azygos vein, Artificial pneumothorax, Case report

## Abstract

Benign tumors of the esophagus are rare. Among them, leiomyomas are common. Surgical enucleation is indicated in cases, which have symptoms or large tumors. The enucleation through video assisted thoracoscopic surgery has been developed as a preferred approach for the majority of lesions in recent years. However, the complete thoracoscopic enucleation for an esophagus leiomyoma at the level of the azygos vein without cutting the vein and nor using artificial pneumothorax by CO_2_ insufflations is a challenge for thoracic surgeons.

This case report was a 64-year-old female who presented dysphagia and chest pain. Chest computed tomography and esophageal endoscopy displayed an esophageal mass. We used complete thoracoscopic enucleation to treat this condition. The tumor was at the level of the azygos vein. Therefore, it was difficult to remove the tumor without cutting the azygos vein without utilizing the artificial pneumothorax. However, we enucleated it completely with no complications.

The complete thoracoscopic enucleation of the esophageal leiomyoma at the level of the azygos vein without cutting the vein without using the artificial pneumothorax should be applied. A methylene blue swallowing study is an alternative method to a barrium swallowing study while the chest tube is still placed in the pleural space.

## 1 Introduction

Benign tumors of the esophagus are rare. Among them, leiomyomas are common. Studies have reported an incidence of benign esophageal tumors ranging from 0.005% to 7.9% in autopsy cases. The true incidence of esophageal leiomyoma is uncertain because many leiomyomas are asymptomatic and are often incidental findings [8]. Esophageal leiomyomas are commonly found in middle-aged male patients with a male to female ratio of 2:1. In 1797, Munro was the first to report an intramural leiomyoma of the esophagus and consequently, Ohsawa performed the first successful surgical enucleation of this type of tumor [4].The thoracoscopic resection of benign esophageal tumors was first described in 1992 [2]. The complete thoracoscopic enucleation for the esophagus leiomyoma at the level of the Azygos vein without cutting the vein and without using artificial pneumothorax by CO_2_ insufflations has been challenging for surgeons. This case report describes a patient with an esophageal leiomyoma at the level of the azygos vein undergoing complete thoracoscopic enucleation successfully at our department of surgery. This case has been reported in line with the SCARE Criteria for case reports [1].

## 2 Case presentation

**Fig. 1:**
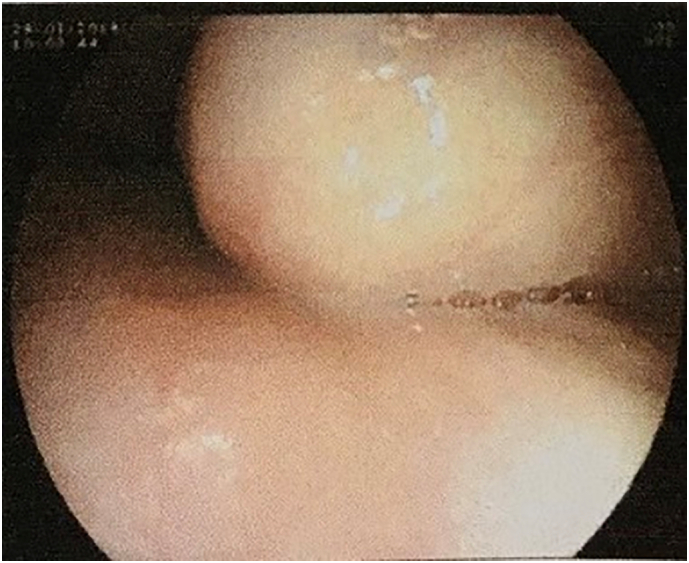
A protrusion into the esophageal lumen on the esophageal endoscopy.

A 64-year-old female was admitted to our hospital due to dysphagia and chest pain persisting for 2 months. The medical history of the patient and her family was not significant. She presented herself at our thoracic department. Results of physical examination were normal. Gastroscopy revealed an esophageal submucosal protrusion with a smooth surface. This mass was about 25 cm from the superior incisor (Fig. 1). We didn't perform endoscopic biopsy because of its benign features on endoscopic imaging.

**Figs. 2, 3:**
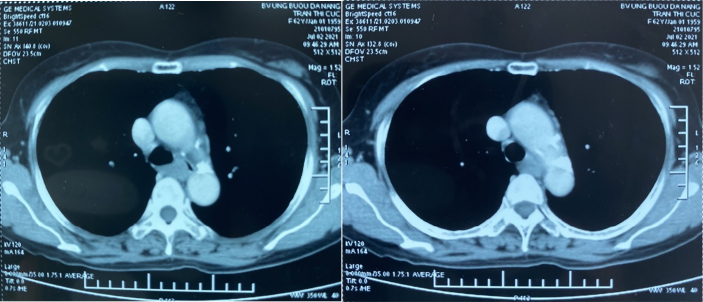
An intramural tumor of the upper third of the esophagus.

The chest computed tomography showed a round high-density shadow on the right edge of the esophagus at the level of the azygos vein (Figs. 2, 3).

**Fig. 4:**
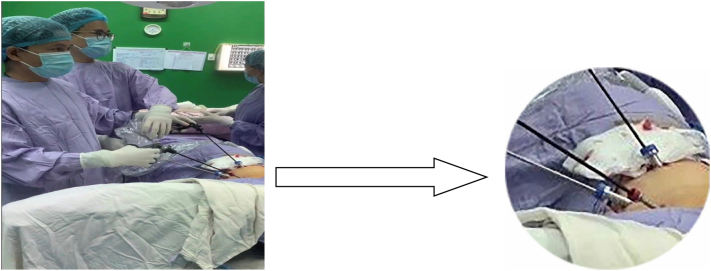
The placement of trocarts, the position of the patient and surgeons.

After preoperative preparation, the complete thoracoscopic enucleation was performed under double-lumen endotracheal intubation of anesthesia. The patient was placed in left lateral decubitus position about 45^0^ frontal incline (semi prone position). The screen was in front of the patient. The surgeon and the assistant surgeon were at the back of the patient. Three trocarts were used. The first trocart of 1 cm in diameter was in the sixth intercostal space at the anterior axillary line for the camera. The second trocart of 1 cm in diameter was in the seventh intercostal space at the posterior axillary line for instruments and devices. The third trocart of 0.5 cm in diameter was in the fourth intercostal space at the middle axillary line for instruments (Fig. 4).

**Fig. 5:**
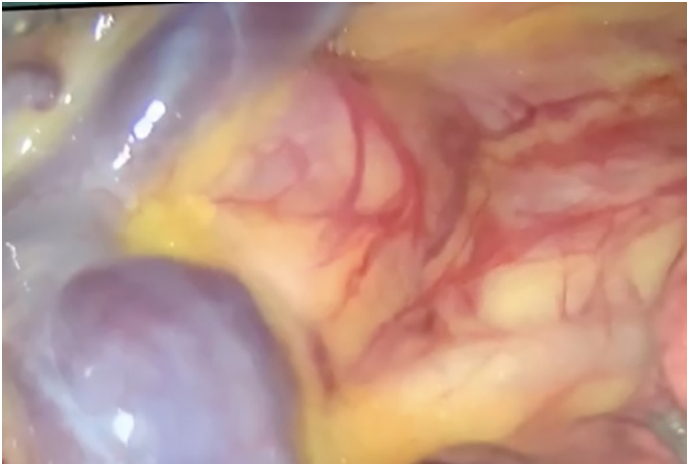
Location of the tumor at the level of the Azygos vein.

We didn't use artificial pneumothorax by CO_2_ insufflation. The thoracoscopic visualisation demonstrated an esophageal intramural tumor at the level of the Azygos vein (Fig. 5).

**Figs. 6, 7:**
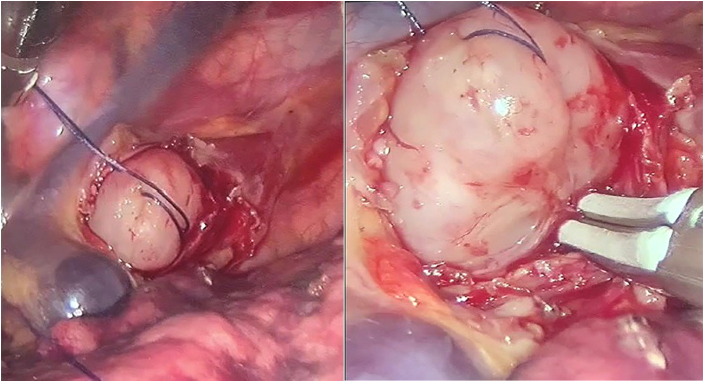
The enucleation of the tumor with a traction suture and without dividing the Azygos vein.

The mediastinal pleura was incised longitudinally above the tumor. The esophageal segment containing the tumor was exposed. The muscle of the esophagus was cut to expose the tumor that has a white, grey color and smooth surface. The tumor was sutured with a vicryl 2.0 to facilitate pulling up the tumor. The tumor was isolated and enucleated completely out of the mucosa. We performed the enucleation of the tumor without cutting the Azygos vein (Figs. 6, 7).

Then we injected 50 ml of air into the esophageal lumen but no bubble of air at the bed of the tumor was recognized. The muscular layer of the esophagus was closed by interrupted sutures of vicryl 3.0. The mediastinal pleura was also sutured (Fig. 6). The tumor was placed in an *endo*-bag and removed out of the thoracic cavity through the incision at the eighth intercostal space at the posterior axillary line. We washed the thoracic cavity by crystal solution. After checking the bleeding at the sites of trocars, a chest tube 28F in size was placed into the pleural space through the incision for the camera. All incisions were sutured by silk. The procedure lasted for 2 h. There were no perioperative complications. The blood loss was minimal.

**Fig. 8:**
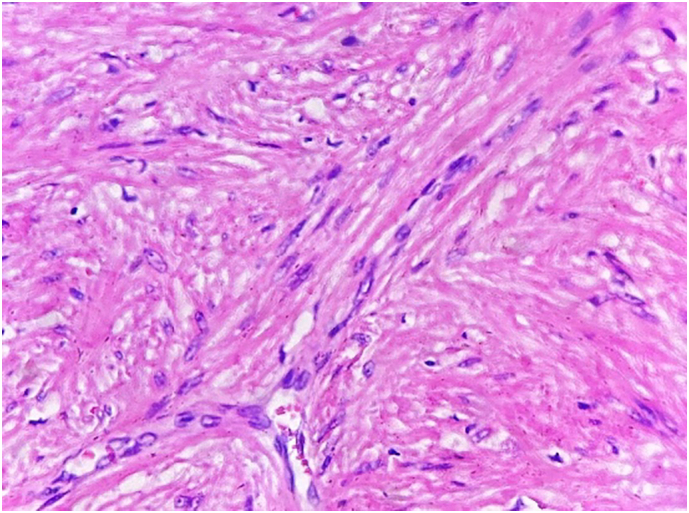
The pathological result of the tumor.

After the operation, the patient was transferred to the surgery intensive care unit. 6 h later, she was transferred to our thoracic surgery department. On the first postoperative day, the patient swallowed 10 ml of methylene blue solution. There wasn't any leakage of the methylene blue in the chest drainage. On the same day, the chest x-ray was normal. Therefore, the chest tube was removed. The patient ate soft food on the third postoperative day and normal diet from the fourth postoperative day. She discharged from the hospital on the fifth postoperative day. The pathological result is leiomyoma (Fig. 8). The immune-histochemical staining was positive for desmin and smooth muscle actin. Furthermore, it was negative for CD 34 and CD 117.

**Figs. 9, 10:**
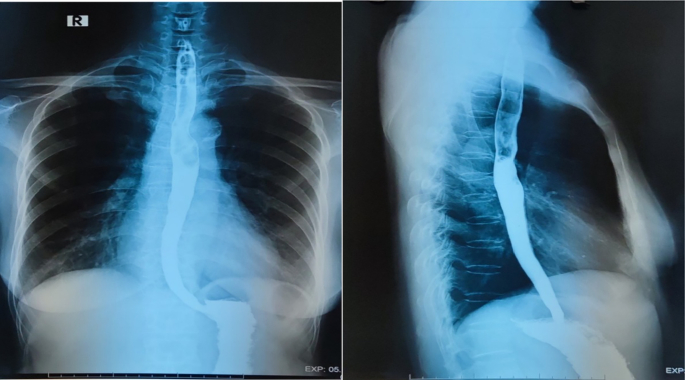
The barium swallowing X-ray at three months of follow up.

During the follow up three months later, the patient didn't have the dysphagia and the vague chest pain. The barium swallowing x-ray was normal (Figs. 9, 10).The patient was satisfied after operation. She stated that she would recommend other patients for the complete thoracoscopic enucleation of the esophageal leiomyoma.

This case was operated by Dr. Trong Vu Than who is the chief of the thoracic surgery department of Da Nang hospital in Vietnam. He is a senior thoracic surgeon who has a vast amount of experience in video assisted thoracic surgery.

Written informed consent was obtained from the patient for publication of this case report and any accompanying images.

## 3 Discussion

Esophageal leiomyoma is more popular than other esophageal benign tumors. It is most commonly located in the middle and lower segments of the esophagus [8]. They are usually found in patients aged from 20 to 50 years old and more often in men than women by a ratio of 2:1 [3]. Lesions are rarely found in the upper third of the esophagus [4]. The patient in our report was a 62- year-old female whose tumor was at the end of the upper third of the esophagus. Milito. P studied 35 patients, between 2002 and 2017, undergoing minimally invasive leiomyoma enucleation. The majority were females. Small tumors are usually asymptomatic. Larger tumors are more likely symptomatic [5]. Dysphagia is by far the most common symptom, followed by retrosternal discomfort and vague chest pain [3]. In this case, the tumor was about 3 cm in longest axis. However, the patient had dysphagia and chest congestion. In our opinion, the tumor is often located in the azygo-esophageal area. This place is narrow, limited by the azygos vein, the aortic arch, the thoracic vertebrae and the heart. Therefore, the tumor locates in the azygo-esophageal area potentially results in compressing on other mediastinal structures and esophageal lumen. As a result, the patient has dysphagia and chest congestion. According to the literature, in cases of suspected leiomyoma, endoscopic biopsy is contraindicated because this procedure involves disruption of the mucosal layer and risks secondary infection, bleeding, and perforation. The incidence of intraoperative mucosal tear is also significantly increased in patients who have undergone preoperative biopsy in the diagnostic workup [4]. We did not perform endoscopic biopsy because of the features of the tumor on the esophageal endoscopy and on the chest computed tomography suggested a benign nature. Traditional options for treatment include observation for small tumors and surgical resection for symptomatic tumors or tumors more than 5 cm in diameter [3]. Enucleation of esophageal leiomyoma has traditionally been performed through thoracotomy [7]. Thoracoscopic enucleation of esophageal leiomyoma was first reported by Everitt in 1992 [2]. Since then, it has been increasingly performed. The thoracoscopic approach offers advantages of being less invasive, avoiding the scarring, and reduced length hospital stay, pulmonary complications and thoracic pain [3]. Jiang. G et al. studied 40 cases of esophageal leiomyoma. They concluded that the enucleation is the first choice for treatment of the esophageal leiomyoma. Thoracoscopic enucleation of esophageal leiomyoma is technically safe and effective. It is currently the best choice for management of esophageal leiomyoma 1 to 5 cm in diameter. It can also be tried on the tumor larger than 5 cm, although the possibility of conversing to thoracotomy increases with huge or horse-shoe shaped tumors [3]. We used complete thoracoscopic enucleation for our patient. Three trocars were placed on the right side of the chest of the patient. The procedure was done under general anesthesia with double lumen intubation. So, we didn't need to use artificial pneumothorax by co2 insufflation. The tumor was sutured with a vicryl 2.0. By grasping this suture, we can lift the tumor out of the submucosa. As a result, we didn't need to divide the azygos vein and the tumor was enucleated easily. In the majority of the upper esophagus tumors, Milito. P divided the arch of the azygos vein [6]. This study is a case report. The size of the tumor was small. So, we performed the procedure without cutting the azygos vein. Moreover, we used the technique of tracing suture on the tumor. This technical skill facilitated the complete enucleation of the tumor without dividing the azygos vein. The technique of placing a traction suture was used by Luh. Sh-P et al. [5], Jiang. G et al. [3]. At the end of operation, the bed of the tumor was submerged in water. The esophageal lumen was insufflated by 50 ml of air. We didn't recognize any bubble of air at the level of tumor. The muscular layer and the mediastinal pleura were closed by some interrupted sutures. There is some disagreement in the literature about whether the myotomy should be sutured after enucleation. Hennessy and Cuschieri stated that a wide breach can be left open without concern, but more experts emphasize the need to reapproximate the muscular wall to prevent mucosal bulging [4]. We agree that the repair of the esophageal wall is necessary to prevent peristatic disorder of the esophagus and pseudodiverticulum after the esophageal enucleation.

On the postoperative day 1, the patient swallowed 10 ml of blue methylene solution to rule out again the leakage of the esophagus. The chest tube was removed on the postoperative day 2. We think the blue methylene swallowing study is necessary instead of doing the barium swallowing study when the chest tube is still in place because of reducing the risk of x-ray exposure to the patient.

## 4 Limitation

This is a case report of a esophageal leiomyoma extending 3 cm in the longest axis. Therefore, we couldn't confirm the feasibility of the complete thoracoscopic enucleation for tumors more than 3 cm at the level of the azygos vein.

## 5 Conclusion

The complete thoracoscopic enucleation of an esophageal leiomyoma at the level of the azygos vein is safe and effective without dividing the azygos vein without using the artificial pneumothorax.

The blue methylene solution swallowing study is an option to rule out the leakage of the esophagus when the chest drainage is still placed in the pleural cavity.

## Declaration of competing interest

The authors have no conflicts of interest to declare.
